# Self-limited single nanowire systems combining all-in-one memristive and neuromorphic functionalities

**DOI:** 10.1038/s41467-018-07330-7

**Published:** 2018-12-04

**Authors:** Gianluca Milano, Michael Luebben, Zheng Ma, Rafal Dunin-Borkowski, Luca Boarino, Candido F. Pirri, Rainer Waser, Carlo Ricciardi, Ilia Valov

**Affiliations:** 1 Department of Applied Science and Technology, Politecnico di Torino, C.so Duca degli Abruzzi 24, 10129 Torino, Italy; 2 Center for Sustainable Future Technologies, Istituto Italiano di Tecnologia, C.so Trento 21, 10129 Torino, Italy; 3 Institute for Materials in Electrical Engineering 2, RWTH Aachen University, Sommerfeldstrasse 24, 52074 Aachen, Germany; 4 JARA – Fundamentals for Future Information Technology, 52425 Jülich, Germany; 5 Ernst Ruska-Centre for Microscopy and Spectroscopy with Electrons Peter Gruenberg Institute Research Centre Juelich, 52425 Jülich, Germany; 6 Nanoscience and Materials Division, INRiM (Istituto Nazionale di Ricerca Metrologica), Strada delle Cacce 91, 10135 Torino, Italy; 7 Peter-Grünberg-Institut (PGI 7), Forschungszentrum Jülich, Wilhelm-Johnen-Straße, 52425 Jülich, Germany

## Abstract

The ability for artificially reproducing human brain type signals’ processing is one of the main challenges in modern information technology, being one of the milestones for developing global communicating networks and artificial intelligence. Electronic devices termed memristors have been proposed as effective artificial synapses able to emulate the plasticity of biological counterparts. Here we report for the first time a single crystalline nanowire based model system capable of combining all memristive functions – non-volatile bipolar memory, multilevel switching, selector and synaptic operations imitating Ca^2+^ dynamics of biological synapses. Besides underlying common electrochemical fundamentals of biological and artificial redox-based synapses, a detailed analysis of the memristive mechanism revealed the importance of surfaces and interfaces in crystalline materials. Our work demonstrates the realization of self-assembled, self-limited devices feasible for implementation via bottom up approach, as an attractive solution for the ultimate system miniaturization needed for the hardware realization of brain-inspired systems.

## Abstract (web summary)

Memristors have become an emerging technology capable in emulating human brain information processing, but understanding and controlling the switching mechanism remains elusive. Here, Milano et al. combine memristive and neuromorphic functionalities in a single crystalline nanowire model system.

## Introduction

Information and communication technologies developed rapidly in the past decade opening new horizons and visions for Green Information Technology, Internet of Things, autonomous/self-learning systems and globally connected networks, all paving the way to artificial intelligence. These new developments challenge the current state-of-the-art nanoelectronics and demand paradigms beyond von Neumann architectures, implementing neuromorphic-type data processing able to emulate human brain functionalities, effectiveness and capacity. Memristive devices based on redox reactions are considered to be one of the most promising candidates for emulating synaptic behaviours and to realize artificial neural networks^1–10^. Obeying same electrochemical fundamentals as biological synapses, redox-based memristors are found to be capable of very similar functionalities. Thus the change of the synaptic connection strength that depends on neuronal electrical signals is emulated by the internal state of resistance (weight) in memristors that is modulated by input electrical stimuli. In biological synapses, synaptic plasticity effects are regulated by Ca^2+^ dynamics^11–13^. Indeed, the rise and subsequent spontaneous decay of Ca^2+^ concentration at the axon terminal caused by electrical neuronal stimuli (action potentials) regulates the release of neurotransmitters into the synaptic cleft. This process is at the root of information processing and memory in the brain. Thus the ability of imitating Ca^2+^ dynamics plays a key role for the realization of bio-realistic artificial synapses. Different thin film devices have been proposed to be able to replicate Ca^2+^ dynamics by using second-order memristors^4,5^ or by exploiting threshold switching properties^6,7^. However, in contrast to biological synapses, the detailed mechanism of operation in memristive synapses is still under debate, suggesting nucleation, diffusion, redox reactions, nanobattery effect and/or combination of these factors as responsible for the observed functionalities^4,6,7,14^. Importantly, all these reports are employing amorphous materials, where often the disordered matter (and deviation of its properties with time) is considered as an internal source of stochastic variability. Single crystalline materials are therefore essential to serve as a model system. Nanowires (NWs) are worldwide exploited as low-dimensional single crystals. Among nanostructures, quasi one-dimensional metal-oxide NWs grown with a bottom–up approach are investigated for the realization of resistive switching devices since they represent an ideal approach for reducing the device size beyond the limits of top–down lithography and can be considered as good platforms for high localization and characterization of the switching events^15,16^. For these reasons, resistive switching was investigated in a wide range of single isolated NWs^15–23^ and NW arrays^24–28^. In addition, by virtue of their high surface-to-volume ratio and unique physical properties, new features of resistive switching characteristics were achieved in NWs by engineering surface electronic properties^26^ or by applying light stimuli^24^. However, single NW-based devices suffer from high operation voltages and/or poor device reliability in terms of endurance and variability since hardware failure due to NW melting can be induced by Joule overheating^15–23^. Moreover, reliable studies concerning the implementation of neuromorphic functions thanks to NW-based memristive devices are currently missing.

Here we demonstrate for the first time a full range of memristive modes showing synaptic/resistive switching functionalities on single NW-based devices. By controlling the formation/dissolution of Ag conductive path along the ZnO NW, we control the operation mode of the NW devices such as bipolar resistive switching with multilevel storage and low switching voltages, selector capabilities characterized by threshold switching and synaptic functions. It was evidenced that Ca^2+^ dynamics of biological synapses are imitated by Ag^+^ ions dynamics on the NW surface, thus demonstrating short-term plasticity (STP) features, such as paired-pulse facilitation (PPF). A detailed investigation of the physical mechanism of synaptic/resistive switching revealed that the classical bulk ionic transport is not playing a significant role, since ionic migration is confined only on the crystal surface. The wide range of functionalities achieved in these devices makes single ZnO NW-based memristors versatile building blocks for nanoelectronics and is a significant step ahead for the realization of NW-based neuromorphic systems.

## Results

### Single NW devices

**Fig. 1:**
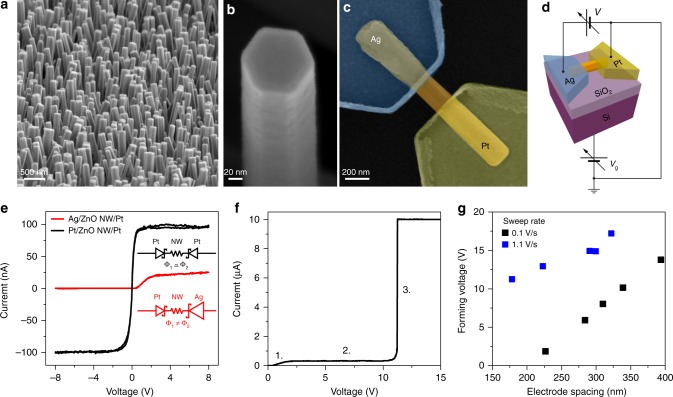
Pristine state and forming process of single ZnO NW-based memristive devices. **a** SEM image of ZnO NW arrays and **b** detail of a vertically aligned and hexagonal-shaped ZnO NW after the growth process. **c** SEM image (in false colours) of a single NW memristive device. The single isolated ZnO NW (orange) is contacted by an Ag electrode (blue) and a Pt electrode (yellow). **d** Schematic representation of the device with electrical connections that includes a back-gate contact for NW field-effect transistor configuration (NW-FET) measurements. **e** Pristine state of single NW-based devices exhibiting back-to-back Schottky diode characteristics. While a symmetric *I*–*V* curve is obtained considering Pt/ZnO NW/Pt devices, the asymmetry of the *I*–*V* curve in case of Ag/ZnO NW/Pt devices has to be attributed to the different energy barriers (Φ) at the Pt/ZnO and Ag/ZnO interfaces. The equivalent circuits composed of back-to-back Shottky diodes with the series resistance of the NW (*R*_NW_) are depicted in the inset. **f** Typical forming curve of Ag/ZnO NW/Pt device characterized by a (1.) diode-like behaviour at low voltage, (2.) current saturation ascribable to the reversely polarized Pt/ZnO junction and (3.) a sharp current transition where the device turns in the ON state. A current compliance of 10 µA was imposed to avoid the full breakdown of the device. **g** Forming voltage dependence on the electrode spacing for two different voltage sweep rates, revealing a substantial reduction of the required forming voltage reducing the electrode spacing and the voltage sweep rate

Single-crystal ZnO NWs were realized with a bottom–up approach by means of a chemical vapour deposition (CVD) synthesis (Methods) that allowed the realization of large area arrays of vertically aligned and hexagonal-shaped ZnO NWs on a Pt substrate^28,29^. The as-synthetized NW morphology investigated by scanning electron microscopy (SEM) is presented in Fig. 1a, b. The median value of length and diameter obtained from their distributions were 1640 and 104 nm, respectively, resulting in an aspect ratio of **~**16 (Supplementary Figure 1). The high chemical purity of as-grown NWs was assessed by X-ray photoelectron spectroscopy (XPS) while X-ray diffraction (XRD) and Raman measurements revealed the high-quality wurtzite crystal structure of NWs (Supplementary Figure 2). Subsequently, single NW-based memristive devices were realized by contacting isolated NWs with Ag and Pt asymmetric electrodes on a SiO_2_ insulating substrate, as presented in Fig. 1c (detail of device fabrication is in Methods). A schematic representation of the device with electrical connections is reported in Fig. 1d.

In order to determine and understand the initial electric properties of the NW devices, the *I*–*V* curves of Pt/ZnO NW/Pt symmetric devices were compared to Ag/ZnO NW/Pt asymmetric devices, as shown in Fig. 1e. In both cases, back-to-back Schottky diode *I*–*V* characteristics are observed^30^, with current saturation for high voltages where the current is limited by the reversely polarized Schottky junction. Pt–Pt symmetrically contacted NWs result in a symmetric *I–V* curve while asymmetric characteristics are observed in case of Ag/ZnO NW/Pt electrodes as a consequence of the different Schottky barrier height at the Pt/ZnO and Ag/ZnO metal–semiconductor interfaces. It is worth noticing that, oppositely to the experimental observations, a lower saturation current is expected when the Pt/ZnO junction is reversely biased because of the higher work function of Pt with respect to Ag. However, the ideal barrier height predicted by the Schottky–Mott rule is strongly influenced by the interface chemistry, as recently discussed by Liu et al.^31^. In our case, the deviation from the ideal behaviour can be explained by the formation of eutectics in between Pt and Zn that are responsible for the formation of interfacial chemical bonds that alter the Pt/ZnO energy barrier height^32^. Thus the maximal electronic current allowed to flow through the system can be controlled by selecting metal electrodes with appropriate work function and interface chemistry. This is an important advantage allowing not only to regulate/limit the absolute electronic current in order to prevent the NW breakdown due to Joule overheating (Supplementary Figure 3) but also to adjust the ionic transference number (the ratio between ionic and electronic partial conductivities), which is an important parameter considering the electrochemical redox reactions and charge transport.

### Electroforming process

The single NW-based device with asymmetric electrodes can be considered as an electrochemical metallization memory cell^33^, where the ZnO NW represents the solid electrolyte while the Ag and Pt electrodes are the electrochemically active and inert electrodes, respectively. When a positive voltage is applied to the Ag electrode, anodic dissolution occurs and Ag^+^ ions start to migrate along the NW towards the Pt counter electrode under the action of the applied electric field. As a consequence, a conductive bridge is formed in between the electrode spacing after reduction and electro-crystallization of metal ions, turning the device in a low resistance state (LRS)^20,21,34^. This electroforming process can be clearly visualized in Fig. 1f where an abrupt increasing of current is observed as a consequence of the metallic conductive bridge formation in between the electrodes. As shown in Fig. 1g, the voltage required to form a conductive channel between the electrodes is strongly influenced by the electrode spacing and the voltage sweep rate, showing that the forming process depends on both electric field and time. High forming voltages can strongly impact the device reliability and the device integration in low-voltage electronics. However, we successfully demonstrate that by properly tuning the device design and applying appropriate voltage sweep rates it is possible to reduce the forming voltage of the NW-based device below 5 V.

**Fig. 2:**
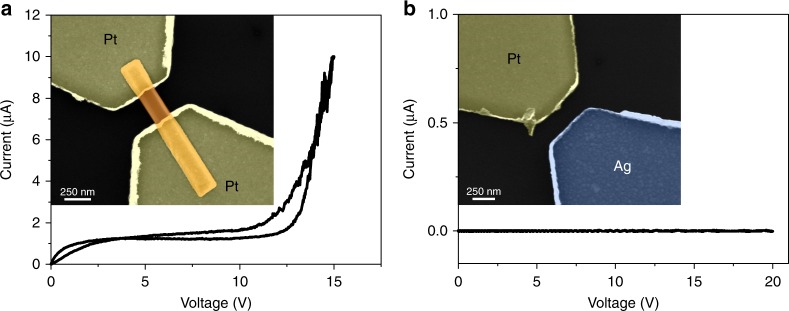
Effect of electrode metal and substrate during electroforming. **a** Typical *I*–*V* curve of a Pt/ZnO NW/Pt device that cannot be formed even for high applied voltages. At higher applied voltages, the current breakdown of the reversely polarized metal–semiconductor junction is observed. The inset shows an SEM image (in false colours) of the Pt/ZnO NW/Pt device. **b** *I*–*V* curve of the control device with Pt and Ag asymmetric electrodes without the presence of the NW that cannot be formed even for high applied voltages. During measurements, the Ag electrode was biased while the Pt electrode was grounded. The resistance of the SiO_2_ substrate was extrapolated to be >10^11^ Ω. The inset shows an SEM image (in false colours) of the device

It is worth noticing that the electroforming cannot be induced without the presence of an electrochemically active electrode (e.g. Ag). Indeed, no forming or switching was observed in Pt/ZnO NW/Pt devices (Fig. 2a).  In planar devices without a NW also no forming/switching has been observed, as reported in Fig. 2b. Thus the switching mechanism can be merely ascribed to the electrochemical migration of metal ions along the ZnO NW (Supplementary Note 1).

### Memristive mechanism

**Fig. 3:**
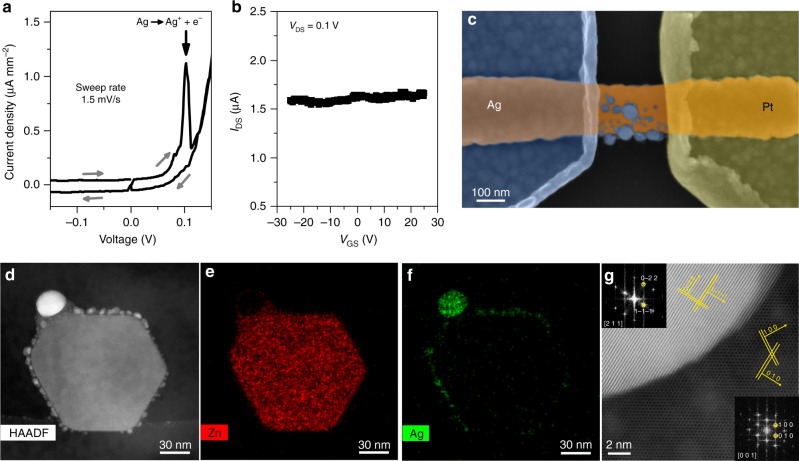
Analysis of the resistive switching mechanism. **a** Cyclic voltammetric measurements of a single NW-based device showing an anodic oxidation peak (indicated by the black arrow) attributable to the Ag oxidation process. Grey arrows indicate the sweeping direction. **b** Transconductance characteristics of the Ag/ZnO NW/Pt FET device in the ON state. No dependence of the drain current *I*_DS_ with respect to the gate voltage *V*_GS_ was observed when the device was in the ON state, suggesting the formation of a metallic-like filament connecting the two electrodes. **c** SEM image (in false colours) of the device after resistive switching showing the presence of an Ag conductive path along the NW. **d** TEM image of a NW cross-section after resistive switching, obtained by cutting the NW in the electrode spacing and relative **e** Zn and **f** Ag EDS maps. Ag nanoclusters can be identified only on the NW surface. **g** HRTEM and corresponding FFT measurements acquired across the interface between the NW and the largest Ag nanocluster showing their crystal structure. Ag and ZnO planes can be clearly resolved; crystallographic directions are indicated by yellow arrows

In order to elucidate the resistive switching mechanism in single NW-based devices, we applied a combined approach using electrical, structural and chemical characterization techniques. The electrochemical reactions preceding the switching events were investigated by cyclic voltammetric (CV) measurements. As shown in Fig. 3a, a redox peak can be clearly resolved at **~**0.1 V by sweeping the voltage at 1.5 mV/s. The peak can be attributed to the anodic dissolution of Ag in analogy with previous observations in thin-film based systems^35^. Instead, no reduction peaks can be detected in the negative voltage region as a consequence of the current limitation due to the Ag/ZnO reversely polarized junction. This experiment demonstrates that CV measurements can be performed also in NW-based resistive switching devices, since the NW behaves as a solid electrolyte at the nanoscale. To gain more insights about the electronic conduction mechanism in LRS (after formation of the conductive path), the transconductance characteristic of the NW in field effect transistor (NW-FET) configuration was measured in the ON state by exploiting the back-gate contact. According to the formation of a metallic conductive path, the drain current did not show any dependence on the gate bias voltage in the ON state as shown in Fig. 3b. After resistive switching, the Ag phase formed along the NW can be directly visualized by SEM, as shown in Fig. 3c. In order to analyze the fine structure of the created conductive path, the cross-section was investigated by cutting the NW perpendicularly to the filament growth direction in between the electrode spacing. Transmission electron microscopic (TEM) and energy-dispersive X-ray spectroscopic (EDS) mapping (Fig. 3d–g) evidenced that a multitude of Ag nanoclusters lies all over the ZnO NW surface while no evidences of the presence of Ag in the NW bulk were observed. This clearly demonstrates that the switching event is highly localized on the NW crystalline surface because of the higher mobility of Ag ions compared to the bulk and the absence of space constrictions. This is an important indication that ionic migration in amorphous films is not following the classical bulk transport but is much faster along nanopores/voids and interfaces. This also well explains the dependence of forming/switching on the oxide film density reported in Cu/Ta_2_O_5_/Pt devices^36^. Even if the diameter distribution of the Ag nanoclusters revealed an average size of 4.5 ± 1.4 nm (Supplementary Figure 4), a larger cluster out of the distribution with average diameter of **~**28 nm can be identified in TEM analyses (Fig. 3d–g). This large Ag cluster, formed on the NW, is likely to play the main role for electronic current conduction in the LRS. Despite a partial reshaping of the NW, the wurtzite crystal structure of the ZnO NW remains preserved all over the NW cross-section after switching, as evidenced by high-resolution TEM (HRTEM) and fast-Fourier transform (FFT) of Fig. 3g that analyses the interface between the large nanocluster and the NW. In addition, we observed that Ag nanoclusters possess face-centred cubic (fcc) crystalline structure.

### Multistate non-volatile switching

**Fig. 4:**
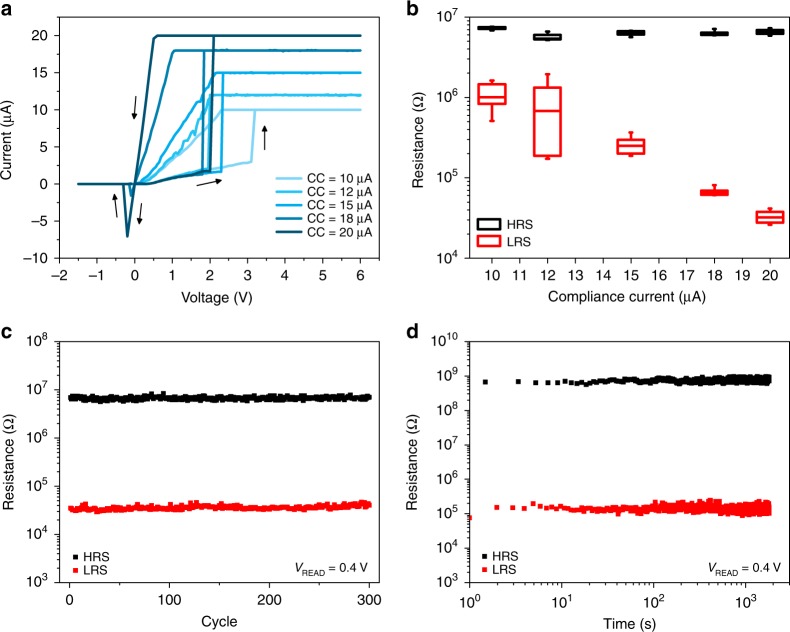
DC operation modes of a single ZnO NW exhibiting multilevel memory capability. **a** *I*–*V* curves obtained by quasi-static DC voltage sweeps for different applied CC, showing bipolar resistive switching behaviour. **b** Measured LRS and HRS resistance values (read at 0.4 V) showing multilevel memory capability through the modulation of the programming current. Box plots were obtained from 15 consecutive cycles for each value of imposed CC on the same device. Midline represent median value, boxes the 25th and 75th percentiles and whiskers the 10th and 90th percentiles. **c** Endurance properties of the device acquired by full-sweep cycles with a programming current of 20 µA and **d** retention properties of a NW-based device after being switched to the LRS and HRS

Direct current (DC) voltage sweep measurements revealed bipolar resistive switching behaviour characterized by low programming voltages (<5 V) and the possibility of accurately controlling the LRS value through the modulation of the compliance current (Fig. 4a). This behaviour arises from the reinforcement of the Ag conductive path with increasing CC^37^. In this fashion, multilevel capability of the NW cell was demonstrated through the modulation of the LRS over much more than one order of magnitude, as presented in Fig. 4b.

The ability of controlling multiple resistance states enable multi-bit memory operations as well as synaptic-weight storage in artificial neural networks. The electrochemical dynamics of these processes are governed by the reorganization of the Ag nanoclusters distribution on the NW surface supported by bipolar electrode effects^38^. Endurance characteristics of the device showed low dispersion and high stability of both LRS and high resistance state (HRS) during 300 full-sweep cycles (Fig. 4c) and retention measurements performed for >10^3^ s are showing no degradation of the resistance state values over time (Fig. 4d).

### Volatile switching and selector capabilities

**Fig. 5:**
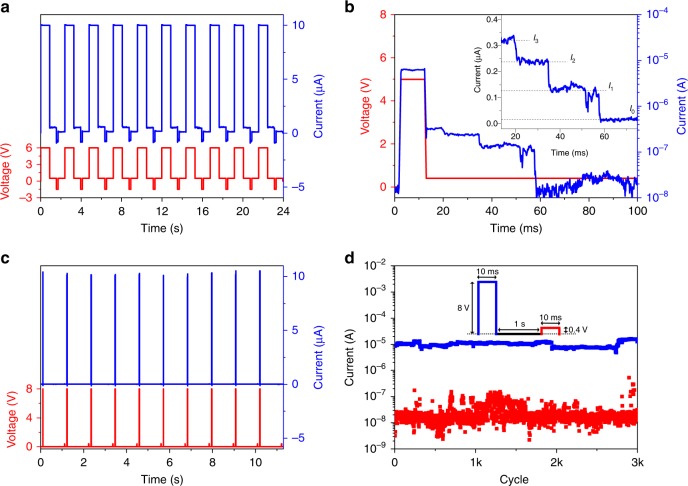
AC operation modes of a single ZnO NW. **a** Non-volatile resistive switching behaviour observed after long writing pulses. Long write pulses (6 V, 880 ms) were applied to SET the device (CC = 10 µA) while erase pulses (−1.5 V, 180 ms) were applied to RESET the device. Current was measured at a reading voltage of 0.5 V. **b** Volatile resistive switching observed after shorter writing pulses. After a relatively short programming pulse (5 V, 10 ms), the device spontaneously relaxes back to the HRS. After pulse, the current relaxation was measured at a reading voltage of 0.4 V. The inset shows the detail of the quantized relaxation process characterized by discrete current steps. **c** Threshold switching behaviour observed by applying short programming pulses. A pulse of 8 V/10 ms was used to program the device while a pulse of 0.4 V/10 ms was used to read the resistance after 1 s. Since the device spontaneously relaxes to the HRS after programming, no RESET pulses are needed. **d** Endurance cycling test properties of the device that exhibited repeatable threshold switching for 3000 cycles. Current data points were sampled during the programming pulse (blue) and during the read pulse (red). The inset shows the applied pulse shape

For hardware implementation of neuromorphic functions, the ability of controlling the resistance states of the NW memristors by applying pulses is crucial. Figure 5a shows that the ZnO NW-based device can be switched between the HRS and LRS by writing (SET) and erasing (RESET) pulses. However, longer writing pulses were necessary to preserve the ON state after programming in order to form non-volatile conductive paths. Indeed, by applying shorter pulses the ON state spontaneously relaxes back to the HRS without needing a RESET, as shown in Fig. 5b. The relaxation process is linked to the spontaneous dissolution of the unstable metallic conductive bridge to form spherical Ag nanoclusters^6,39^. As shown in Fig. 5b, the progressive disconnection of Ag nanoclusters from the metallic filament resulted in a relaxation process with discrete (Landauer) conductance values. The driving forces of the relaxation process is the nanobattery effect including minimization of the interfacial energy (Gibbs–Thomson effect)and electromotive force effects induced by Nearnst potential and diffusion potential^6,14,39,40^. For these reasons, short programming pulses result in volatile threshold switching behaviour of the device, as can be observed in Fig. 5c. This repeatable threshold switching over cycling and high current non-linearity (Fig. 5d) make these devices promising for the realization of selectors, useful for solving the sneak path problem in crossbar architectures^40^. In addition, we report that the output current during threshold switching can be modulated by changing the programming pulse amplitude (Supplementary Figure 5). To our best knowledge, these results represent the first evidence that single NWs can successfully act as selector devices.

### Neuromorphic functionalities and artificial synapse

**Fig. 6:**
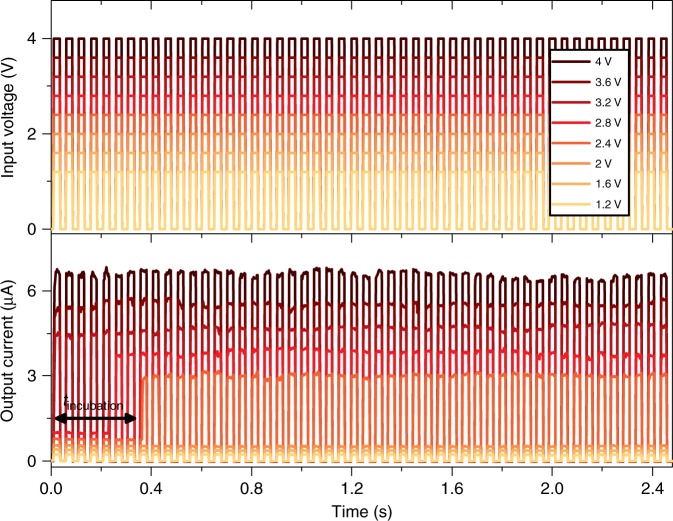
Single NW device response to an input train of electrical pulses. The higher panel shows the input voltage pulse trains composed of 50 pulses of 22 ms length (22 ms of waiting time) for different voltage amplitudes (from 1.2 to 4 V). The current responses of the device are presented in the lower panel, showing current jumps for voltage amplitudes ≥2.4 V. The number of pulses needed for inducing a switching event (i.e. the incubation time) decreases by increasing the pulse amplitude. The incubation time observed by stimulating the device with a pulse train of 2.4 V amplitude is indicated by the arrow

Another needed feature for neuromorphic hardware is the implementation of electric train pulses as input stimuli. The device response to such stimuli is highly dependent on the number of applied pulses as well as on their amplitude. In Fig. 6, the output current of the single NW device is recorded during the application of voltage pulses with a length of 22 ms, using various voltage amplitudes. Below a certain threshold voltage amplitude (≤2 V), pulse trains do not result in a change of the device conductivity. However, for higher voltages, current jumps were observed after a certain amount of applied pulses (incubation time). The incubation time can be shortened by increasing the voltage pulse amplitude. Finally, for high pulse amplitude (≥3.2 V), the switching events occur during the first applied pulse. It is important to note that both the minimum of voltage pulse amplitude required for switching as well as the incubation time can be modulated/controlled by altering the pulse length (Supplementary Figure 6).

**Fig. 7:**
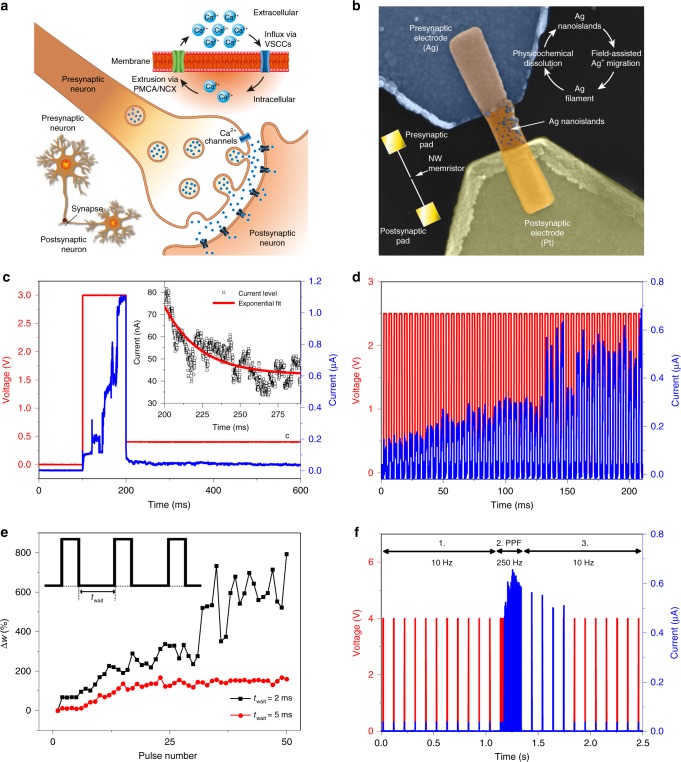
Short-term synaptic plasticity of single ZnO NW device. Similarities between **a** Ca^2+^ dynamics in biological synapses and **b** Ag^+^ dynamics on the single ZnO NW surface. The Ag filament formation and subsequent spontaneous dissolution in Ag nanoclusters can emulate the influx and extrusion of Ca^2+^ from the neuron cell. **c** Gradual increase of the device conductivity observed by applying a long pulse (3 V, 100 ms) and subsequent device relaxation observed at a reading voltage of 0.4 V. The inset shows a detail of the spontaneous device relaxation to the ground state (HRS) after the electrical stimulus. Current relaxation can be well interpolated by an exponential decay function. **d** Experimental demonstration of PPF in a single NW device. In blue, the current response of the device to multiple input voltage pulses (2.5 V, 2 ms pulses, 2 ms waiting time). **e** Percentage of conductance change (weight) as a function of pulse number (2.5 V, 2 ms pulses) for different waiting time in between pulses. Higher waiting time result in lower percentage of conductance change. **f** (1.) Low frequency short pulses (4 V, 2 ms pulses, 10 Hz) are not sufficient for increasing the device conductance, whereas (2.) PPF is observed by increasing the pulse frequency (4 V, 2 ms pulses, 250 Hz, 50 pulses). (3.) After PPF, the device relaxed back to the initial state when the pulse frequency is again lowered to 10 Hz

Thus it is evident that a certain amount of time and pulse amplitude is required to form a conductive path between electrodes. By properly adjusting these parameters, the Ag dynamics on the NW surface driven by short trains of electrical pulses can be exploited to emulate Ca^2+^ dynamics underlying synaptic plasticity of biological synapses, as depicted in Fig. 7a, b. Chemical synapses, which connect pre-neurons to post-neurons, play a key role in higher brain functions, such as memory and learning. All these processes are regulated by synaptic plasticity that consists of change of the strength of the synaptic connection (synaptic weight) between pre- and post-neurons^13^. Electrical stimuli (action potentials) reach the presynaptic terminal and lead to the opening of voltage-dependent Ca^2+^ channels^41^. This leads to an influx of Ca^2+^ ions inside the neuron terminal with consequent release of neurotransmitters in the synaptic cleft and enhanced synaptic transmission^41^. After that, the basal intracellular Ca^2+^ concentration is restored by extrusion of Ca^2+^ ions out of the cell by means of plasma membrane transport proteins, such as Ca^2+^ ATPase and Na^+^/Ca^2+^ exchanger^42^. In a similar way, electric field induced by voltage pulses causes Ag^+^ ion migration on the NW surface forming a conductive bridge in between electrodes with consequent enhancement of the device conductivity. As presented in Fig. 7c, a, low-voltage electrical stimulus applied to the presynaptic electrode (3 V, 100 ms) leads to a gradual step-wise increase of the device conductivity, due to the progressive migration and rearrangement of Ag^+^ ions with consequent reinforcement of the conductive path in between electrodes (Supplementary Note 2). After the electrical stimulus, the device spontaneously relaxes back to the ground state thus emulating the Ca^2+^ extrusion process, with a current relaxation characteristic that can be well interpolated by an exponential decay function (inset of Fig. 7c). The time constant (*τ*_r_) of the NW-based device (~23 ms) is on the same order of biological synapses where STP acts from milliseconds to minutes^13^. Note that the kinetics of the conductive bridge growth and relaxation on the NW surface can be influenced by both pulse amplitude and length^6,7^ as well as temperature^43^ and moisture^44^. Similarities between Ag^+^ and Ca^2+^ dynamics allow the single NW memristor to emulate some typical behaviours of STP in biological synapses. The application of short paired pulses applied within a short interval to the presynaptic pad results in a gradual increase of the NW conductance as the number of pulses increases as shown in Fig. 7d, emulating PPF^13^. Note that after PPF the device spontaneously relaxes back to the ground state (Supplementary Figure 7). This behaviour is a consequence of the interplay between the Ag conductive channel reinforcement driven by the electric field during the pulse application and physicochemical dissolution during waiting time in between pulses. As shown in Fig. 7e, this results in a higher percentage of conductance change (weight) when the waiting time in between pulses is reduced. For a waiting time between pulses longer than the relaxation time characteristic, no variation of the percentage of conductance change was observed. The conductance change dependence on the spiking frequency can be clearly observed in Fig. 7f. Here low frequency pulses are not sufficient to induce a variation of the conductance (1.), whereas PPF was observed by increasing the pulse frequency (2.). The subsequent application of low frequency pulses results in a relaxation of the device to the ground state (3.), evidencing that low spiking frequencies are not sufficient for sustaining the high conductive state induced by high frequency spiking.

## Discussion

ZnO single NW-based devices were used as model system to study processes and behaviour of memristive systems, demonstrating bipolar switching, selector functions and reliable emulation of short-term plasticity of biological synapses. It was shown that proper selection of electrode/electrolyte materials combination and operating conditions determine device properties and are key parameters to successfully control and switch between these different types of memristive functionalities. The underlying mechanism of filament formation/dissolution has been studied in detail and discussed in the light of electrochemical fundamentals, highlighting the Ag^+^/Ag redox reactions and transport dynamics on the crystalline NW surface. It was demonstrated that the device conductance can be modulated by repeated pulses applied within a short interval. The progressive increase of device conductance during electrical stimulation and subsequent spontaneous decay over time can well emulate the Ca^2+^ dynamics of biological synapses, a key process for the realization of bio-realistic artificial synapses. These observations suggest that single NW devices can be implemented as reliable building units for the realization of artificial synapses based on self-assembled nanostructures grown with a bottom–up method, a promising approach for device scaling, being an essential milestone on the way for the realization of NW-based artificial neural networks.

## Methods

### NW synthesis and characterization

ZnO NWs were synthetized in a horizontal tubular furnace by low-pressure CVD (LP-CVD), as previously reported^28,29^. A Pt thin film (100 nm) was realized by sputtering (Kurt J. Lesker, PVD 75) on a SiO_2_ (190 nm)/Si commercial wafer by using a Ta (20 nm) adhesion layer. The Pt film was used as catalyst and was placed into the quartz tube of the furnace on an alumina boat, surrounded by a Zn foil (purity 99.99 %) that acted as the Zn source. LP-CVD was performed at 650 °C fluxing 200 sccm of O_2_ as gas precursor and 300 sccm of Ar as carrier gas into the chamber (pressure of 1.6 Torr). The morphology of as-grown nanostructures was investigated by field-emission SEM (FE-SEM; Zeiss Merlin). Chemical composition of as-grown ZnO NW arrays was assessed by means of XPS, using a Kα source with energy of 1486.6 eV and using C 1s peak position (284.8 eV) as calibration. XPS high-resolution peaks were interpolated by means of Gaussian–Lorentzian functions after Shirley’s background subtraction. In addition, the structural properties were investigated by XRD in Bragg–Brentano theta-2 theta configuration and by Raman spectroscopy performed with a Renishaw inVia Reflex micro-Raman spectrophotometer equipped with a cooled charge-coupled device camera and by using an excitation wavelength of 514 nm. Raman peaks were interpolated by Lorentzian functions after background subtraction.

### Single NW device fabrication

Single ZnO NW devices were fabricated by combining optical and e-beam lithography (EBL). First, optical lithography was used to pattern a sub-millimetric probe circuit using a customized optical mask on a SiO_2_(450 nm)/Si (p-type, B doped) substrate. Pads and probe paths geometries were thus realized by sputtering deposition of Ti/Au followed by a lift-off process in acetone. ZnO NWs were then mechanically transferred from the growth substrate to a ~120 × 120 μm^2^ selected area of the probe circuit using a mounted hair and a optical microscope. The NW position on the substrate was referenced by using proper markers and SEM imaging (SEM; FEI Inspect F™). Subsequently, sub-micrometric paths connecting the probe circuit to single selected NWs were defined by EBL (FEI Quanta™ 3D Microscope) followed by 80 nm of metal deposition by sputtering (Kurt J. Lesker, PVD 75) and lift-off in acetone. Two EBL processes were performed in order to obtain devices with asymmetric Ag and Pt electrodes. In order to improve the contact reliability, the sample was treated with oxygen plasma (40 W, 30 s) before metal deposition. The NW-FET configuration for transconductance measurements was obtained by contacting the p-type Si back-gate with Ag conductive paste while the SiO_2_ layer acted as the gate dielectric. Note that, during the whole fabrication process, the device exposure to aqueous solutions was strictly avoided in order to preserve ZnO NWs from degradation, as we have investigated in our previous work^29^. In order to analyse the resistive switching mechanism and the change in the device morphology after the application of an electric field, the devices were investigated by SEM (FE-SEM; Zeiss Merlin).

### Electrical characterization

DC and alternating current (AC) electrical measurements were performed in two terminal configurations, biasing the Ag electrode while the Pt electrode was grounded. Pristine state characterization as well as CV measurements were performed by means of a Keithley 6430 sub-femtometer sourcemeter with remote preamplifier. A device with high electrode spacing (385 nm) was realized for measuring the pristine state of the Ag/ZnO NW/Pt device in a wide voltage range without inducing the device electroforming. In CV measurements, the current density was calculated by considering the NW section area extrapolated from SEM analyses. Transconductance characteristic of the Ag/ZnO NW/Pt device in the ON state performed in FET configuration was measured by means of the Keithley 6430 sub-femtometer sourcemeter coupled with a Keithley 2410 used to sweep the gate bias voltage. Transconductance curve was measured in quasi steady-state conditions by sweeping the gate bias voltage at 0.2 V/s and applying a constant drain-source bias. The gate leakage current was measured to be negligible during measurements. DC characterization and long pulse measurements (measurements in Figs. 4, 5a and 6) were performed by means of a Keithley 2636A and a customized probe station. Short pulse measurements (device relaxation, selector properties and neuromorphic measurements in Figs. 5b, c, d and 7) were performed by means of a Keithley 4200 semiconductor device analyser equipped with a Pulse Measuring Unit and a SemiProbe probe station with a resistance in series to the device in order to limit the maximum flowing current (330 kΩ, 1 MΩ in Fig. 7d, e). The conductance relaxation characteristic of the device was interpolated by the exponential decay function *I*(*t*) = *I*_0_ + *C*exp(−*t*/*τ*_r_), where *I*_0_ represents the ground state current, *C* a pre-factor constant and *τ*_r_ the relaxation time constant. The change of the memristor conductance weight (Δ*w*) was calculated as Δ*w* = [*I*(*n*) − *I*(1)]/*I*(1), where *I*(*n*) is the current flowing into the device during the *n*^th^ pulse of the pulse train. All electrical measurements were performed in air at room temperature.

### Sample preparation for TEM

A FEI dual-beam FIB Helios Nanolab 460F1 workstation was employed for the cross-section sample preparation. A carbon layer was deposited on the field of interest using electron beam to protect the sample surface, then a Pt layer was deposited using Ga ion beam for further protection. “Lift-out” technique was used to prepare TEM specimen. Ga ions were accelerated at 30 kV and the whole Ion milling process was finished in <30 mins; both the energy and time are not enough for inducing morphological changes of the sample. The specimen was additionally polished with a Fischione’s Model 1040 NanoMill system to reduce surface roughness and eliminate contaminations.

### TEM characterization

A FEI Titan G2 60–200 ChemiSTEM microscope equipped with a spherical aberration corrector for the probe forming system and Super-X EDS was used to acquire high-angle annular dark-field images and to perform elemental analysis. The microscope operated at 200 kV, HRTEM images provide direct information about the atomic structure and EDS mapping shows the element distribution of the sample.

## Electronic supplementary material

Supplementary Information

Peer Review File

## Data Availability

The data that support the findings of this study are available from the authors on reasonable request; see Author contributions for specific data sets. The Source Data underlying Fig. 4b are provided as a Source Data file.
