# Supplementary material for: Self-limited single nanowire systems combining all-in-one memristive and neuromorphic functionalities
Source: Nat Commun. 2018 Dec 4;9:5151. doi: 10.1038/s41467-018-07330-7 (PMC6279771; doi:10.1038/s41467-018-07330-7)
Supplement: Supplementary file 1 — Supplementary Information [file 41467_2018_7330_MOESM1_ESM.pdf]

Self-limited single nanowire systems combining all-in-one memristive and neuromorphic functionalities– Supplementary Information

Gianluca Milano[1,2], Michael Luebben[3,4], Zheng Ma[5], Rafal Dunin-Borkowski[5], Luca Boarino[6], Candido F. Pirri[1,2], Rainer Waser[3,4,7], Carlo Ricciardi*[1] and Ilia Valov*[4,7]

[1]Department of Applied Science and Technology, Politecnico di Torino, C.so Duca degli Abruzzi 24, 10129 Torino, Italy.

[2]Center for Sustainable Future Technologies, Istituto Italiano di Tecnologia, C.so Trento 21, 10129 Torino, Italy.

[3]Institute for Materials in Electrical Engineering II, RWTH Aachen University, Sommerfeldstrasse 24, 52074 Aachen, Germany.

[4]JARA – Fundamentals for Future Information Technology, 52425 Jülich, Germany.

[5]Ernst Ruska-Centre for Microscopy and Spectroscopy with Electrons Peter Gruenberg Institute Research Centre Juelich, 52425 Jülich, Germany.

[6]Nanoscience and Materials Division, INRiM (Istituto Nazionale di Ricerca Metrologica), Strada delle Cacce 91, 10135 Torino, Italy.

[7]Peter-Grünberg-Institut (PGI 7), Forschungszentrum Jülich, Wilhelm-Johnen-Straße, 52425 Jülich, Germany.

*e-mails: carlo.ricciardi@polito.it; i.valov@fz-juelich.de

Supplementary Figures

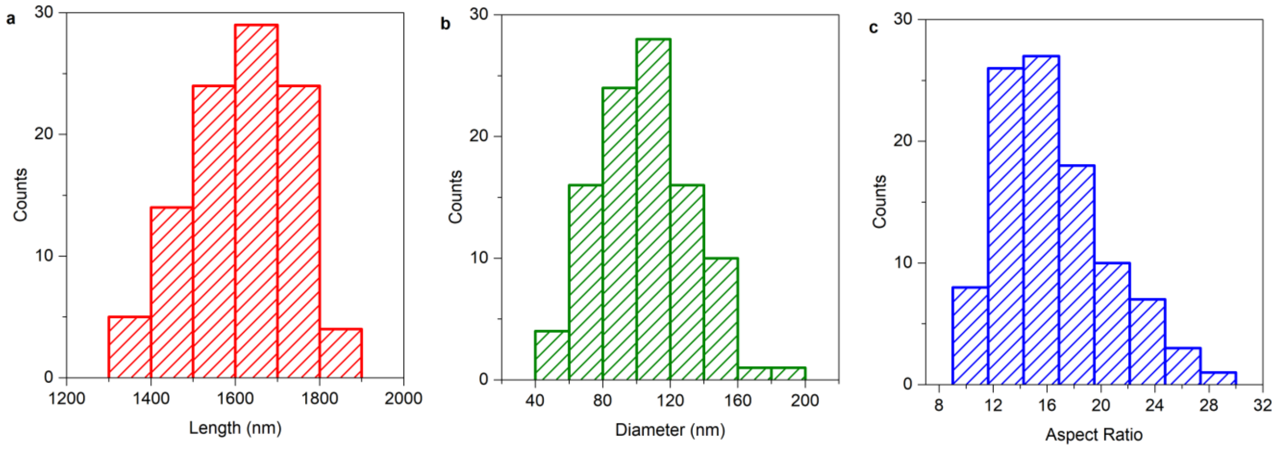

Supplementary Figure S1 | Length, diameter and aspect ratio distribution of ZnO NWs synthetized by CVD. a, NW length distribution, **b,** diameter distribution and **c,** aspect ratio distribution obtained by measuring 100 NWs in cross-sectional SEM images acquired in different areas of the sample.

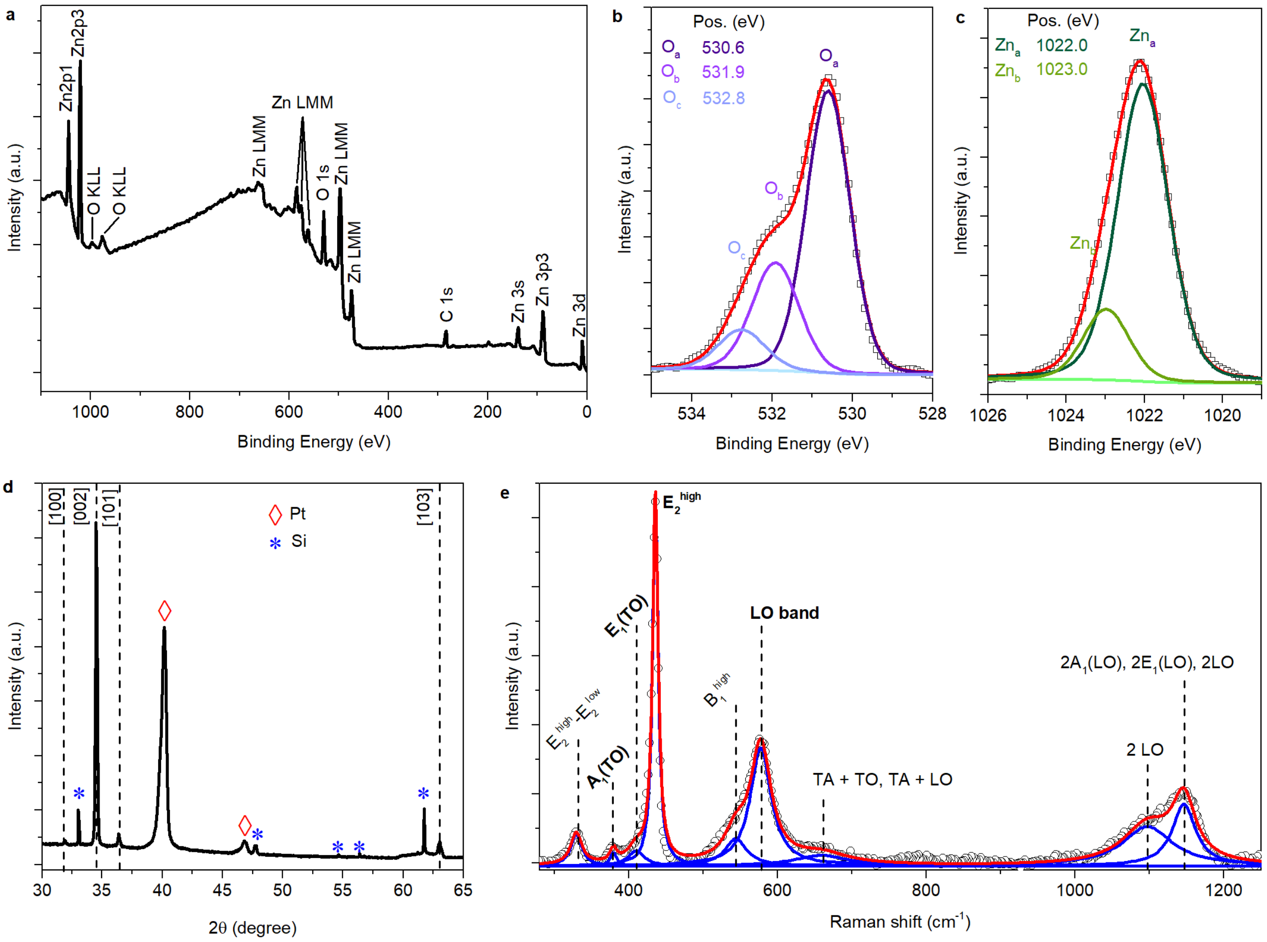

Supplementary Figure S2 | Chemical and structural characterization of ZnO NWs. a, XPS survey spectra of as grown ZnO NW arrays after synthesis revealing high chemical purity of as grown nanostructures with the presence of Zn and O peaks. The C peak can be attributed to contaminants present on the surface as a consequence of sample exposure to atmospheric conditions[1]. **b,** High-resolution spectra of O 1s core level that can be interpolated by means of 3 components: O_a can be attributed to O^{2-} ions in Zn-O bond, O_b to O^{2-} in oxygen deficient regions and O_c to hydroxyl groups. **c,** High-resolution spectra of $Zn2p_{3/2}$ level interpolated by 2 components: Zn_a is attributable to Zn-O bonds while Zn_b to the hydrated layer on the NW surface. Open squares are raw data while red line represent the cumulative fit peak. Data were analysed accordingly to our previous work[1]. **d,** XRD pattern of as grown ZnO NW arrays that match well with the ZnO wurtzite crystal structure with $P6_{3mc}$ symmetry. ZnO peaks are labelled according to

the standard data file JCPDS No. 89-0511. Comparing the relative intensities of XRD peaks with intensities from the standard data file for ZnO powder, it can be noticed that NWs are highly oriented along the [002] direction. This is a direct consequence of the NW growth along the c-axis direction. **e,** Raman spectra of ZnO NWs. Open circles represent background subtracted data while red curve represents the cumulative fit and blue curve the single components of the fit. Peaks relatives to a ZnO wurtzite crystal structure are labelled according to Cusco et al.[2]; first-order Raman peaks are labelled in bold. According to Zhang et al.[3], the narrow E_2^{high} peak (FWHM<10) indicate high crystal quality. A detailed discussion of Raman spectra of ZnO NWs can be found in our previous work[4].

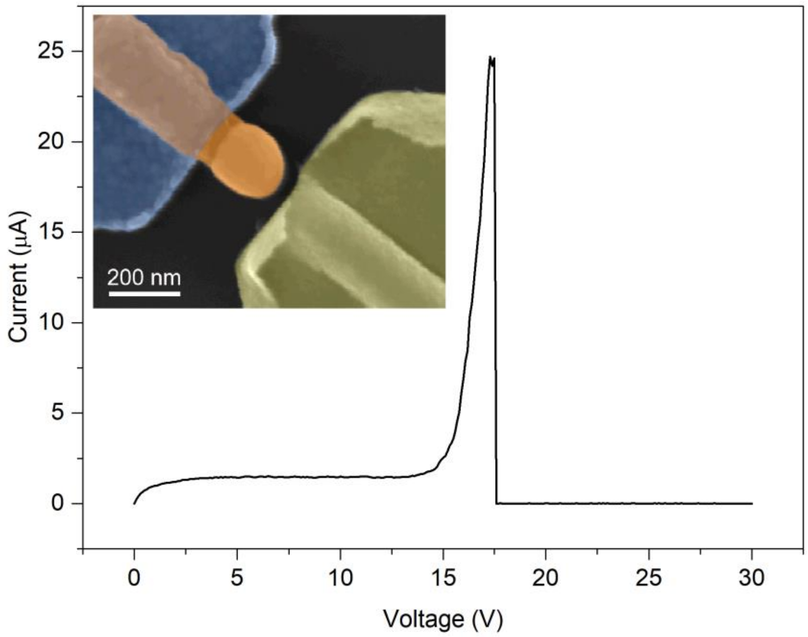

Supplementary Figure S3 | Hard breakdown of the ZnO NW due to Joule overheating. *I-V* curve of a Pt/single ZnO NW/Pt device exhibiting hard breakdown of the device. The device failure occurs only for high applied voltages (> 15 V) since the current flowing into the device (and consequent Joule overheating) is previously self-limited by the inversely polarized Pt/ZnO junction. After the self-limited regime, the breakdown current of the junction was observed and, when the critical value of ~ 25μA was reached, a dramatic drop of current can be observed. After this irreversible hard failure of the NW, the NW-based device resulted to be an open circuit as a consequence of the NW melting due to Joule heating. Note that the evaluation of the NW breakdown process was performed by considering a symmetrically Pt contacted NW in order to avoid any effects due to migration of the Ag electrochemically active electrode during the application of high electric fields. The inset shows an SEM image (in false colors) of an Ag/ZnO NW/Pt memristive device after hard breakdown due to Joule overheating. The current self-limitation (in the operational voltage window of resistive switching) is crucial during the forming step and as a protection for overshooting, playing a key role for the device reliability. Otherwise, the NW melting due to Joule overheating turns the device irreparably to an HRS, resulting in a permanent device failure.

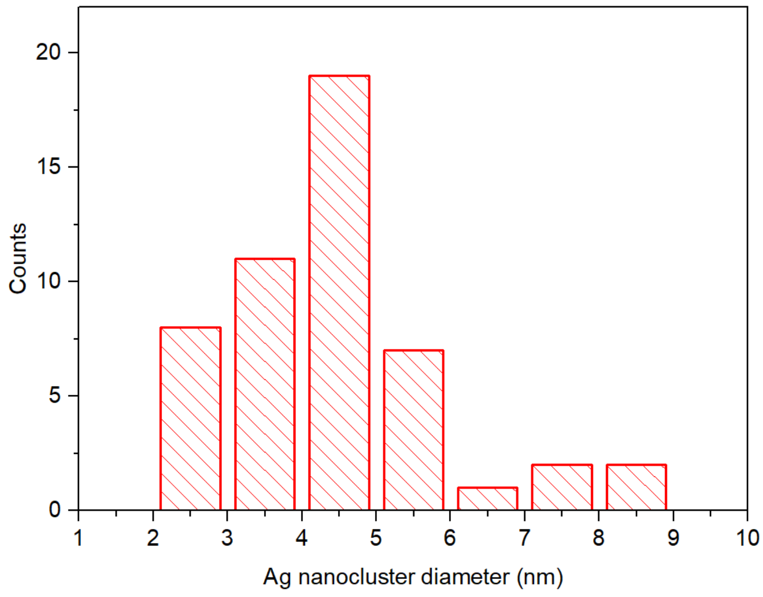

Supplementary Figure S4 | Ag nanocluster size distribution. Histogram of diameters of Ag nanoclusters lying on the NW surface after resistive switching. The diameter distribution was obtained by measuring nanocluster sizes in cross-sectional TEM image. The mean diameter value is (4.5 ± 1.4) nm while the median value is 4.4 nm.

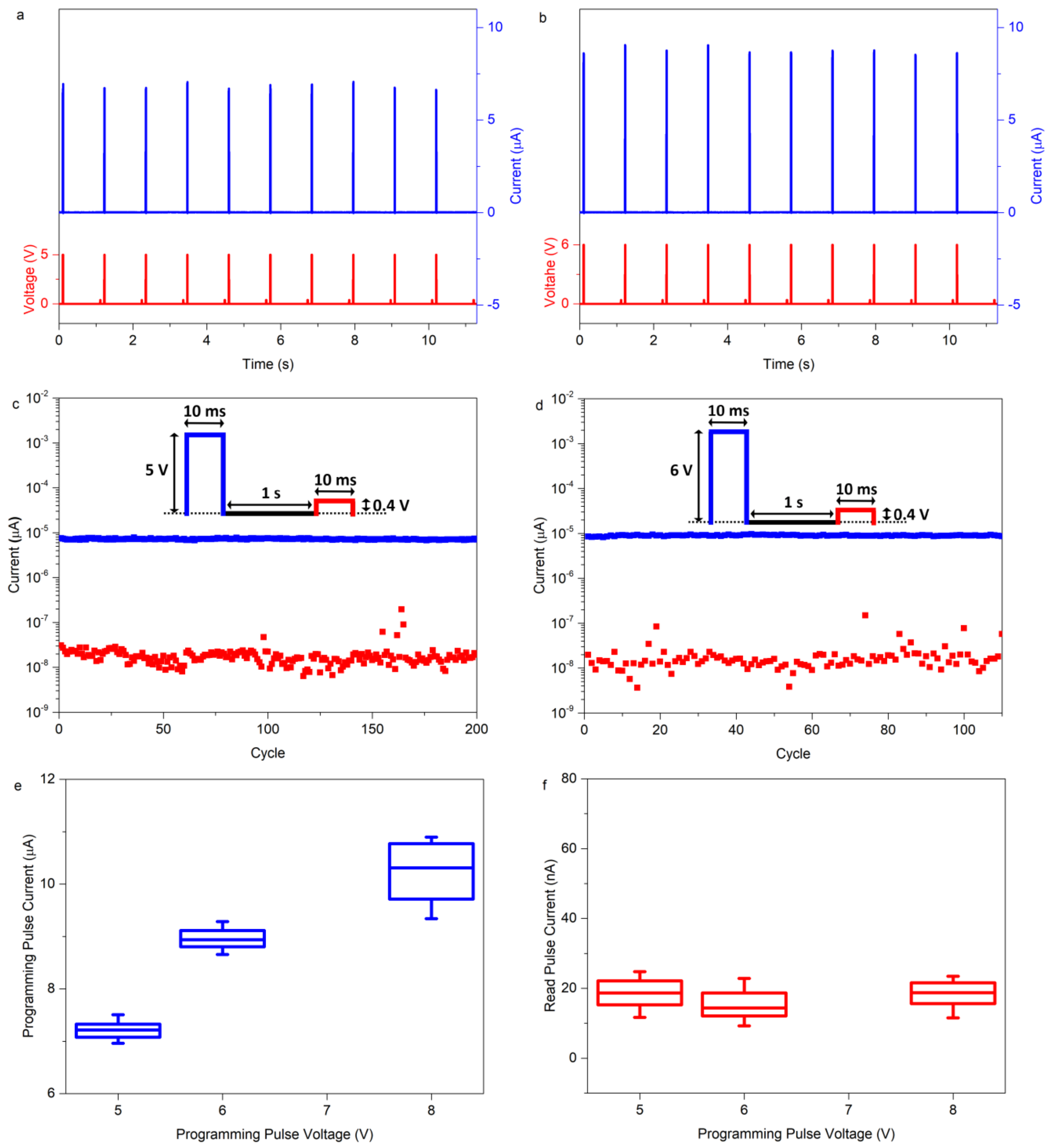

Supplementary Figure S5 | Single NW selector device – output current dependence on programming pulse. Threshold switching behaviour of the single NW selector device by applying a programming pulse of 10 ms with amplitude of **a,** 5 V and **b,** 6 V. Endurance cycling test of the device with a programming pulse of **c,** 5 V and **d,** 6 V showing good stability for each programming pulse amplitude. Current data points were sampled during the programming pulse

(blue) and during the read pulse (red). The inset shows the applied pulse shape. **e,** Current response of the selector device during the programming pulse depending on the programming pulse amplitude. Higher programming pulse resulted in higher output current. **f,** Current response of the device during the read pulse performed after 1 s from the programming pulse as a function of the programming pulse amplitude. In all cases, the device resulted to be relaxed to the HRS during the read pulse. Boxplots were created by considering 100 consecutive cycles. Midline represent median value, box the 25th and 75th percentiles and whiskers the 10th and 90th percentiles.

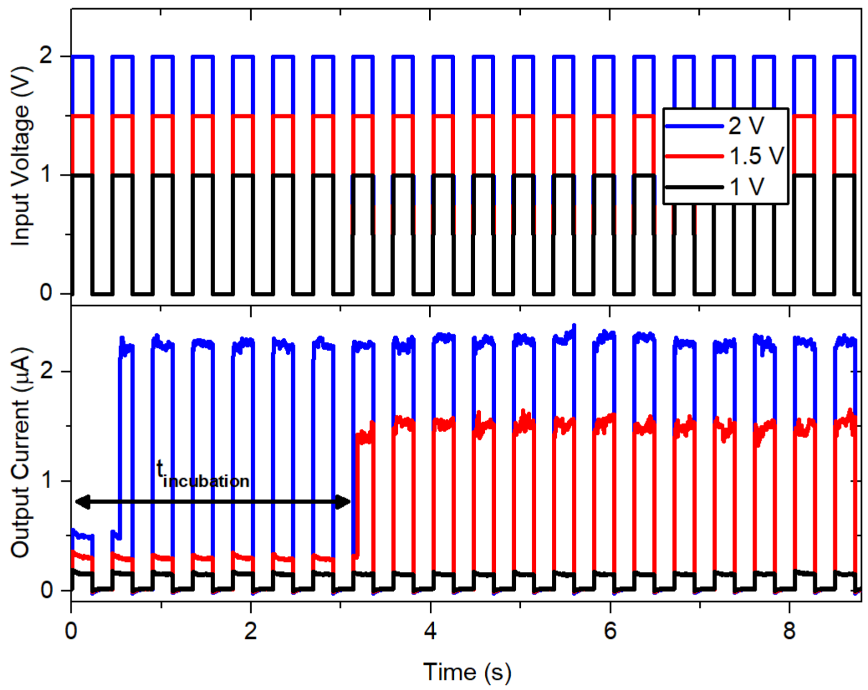

Supplementary Figure S6 | Single NW-device response to an input train of electrical pulses with extended length and long waiting time in between pulses. The higher panel shows the input voltage pulse trains of 20 pulses of 220 ms length (220 ms of waiting time) for different voltage amplitudes (1, 1.5 and 2V). The current responses of the device are presented in the lower panel, showing current jumps for voltage amplitudes ≥ 1.5 V. The number of pulses (i.e. the incubation time) needed for inducing a switching event decrease by increasing the pulse amplitude. These results, compared to results in Fig.5, showed that the use of lower frequency square waveforms allows switching the device thanks to a lower number of pulses. The incubation time observed by stimulating the device with a pulse train of 1.5 V amplitude is indicated by the arrow.

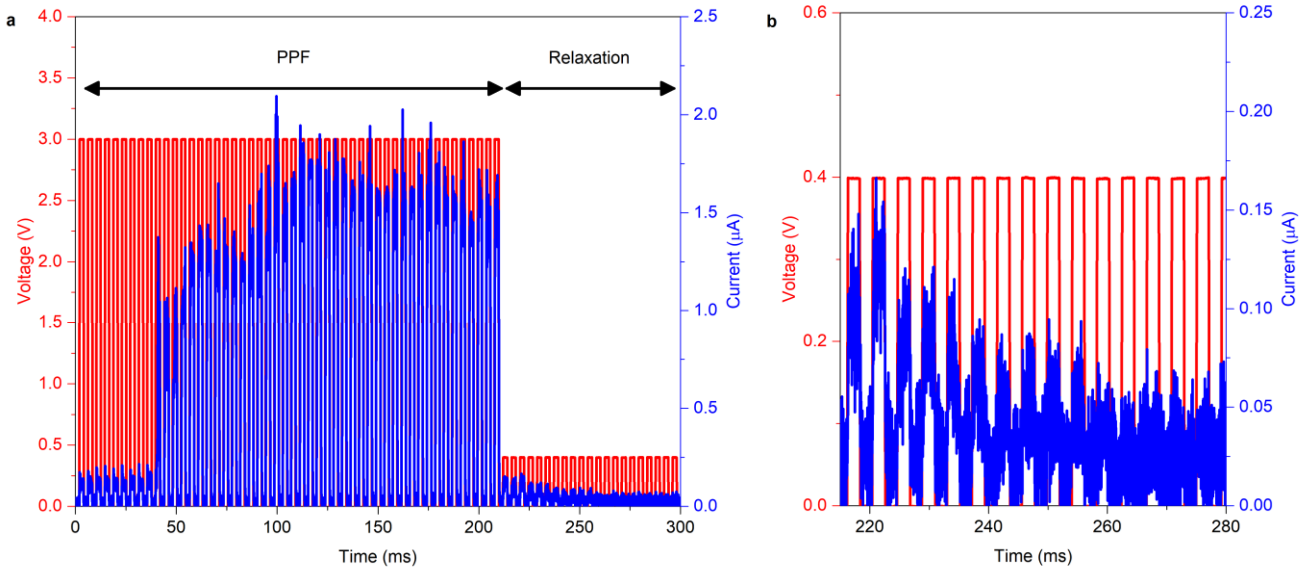

Supplementary Figure S7 | Single NW-device response to an input train of voltage pulses followed by lower amplitude read pulses. a, After the device excitation by subsequent pulses (PPF) (3 V, 2 ms), the device spontaneously relaxes back to the initial ground resistance state as investigated by device interrogation of low voltage read pulses (0.4 V, 2 ms). **b,** Zoom of the relaxation process after PPF, showing in details the conductivity relaxation to the ground state with a finite relaxation time in the order of tens of milliseconds.

Supplementary Notes

Supplementary Note 1:

A wide range of physical mechanisms were proposed in order to explain resistive switching mechanism observed in ZnO NWs and nanorods (NRs).[4–14] Resistive switching in ZnO NRs arrays was already reported in 2010 by Chang et al.[5]. In this case, the switching mechanism was ascribed to the formation of conductive filaments on the NW surface composed of oxygen vacancies and/or Zn interstitials. However, the switching mechanism in capacitor-like devices composed of NW and NR arrays can differ from the mechanism underlaying resistive switching in a single isolated NW because of the different structure of the active layer and the metal electrode morphology. We have recently proposed an innovative understanding of the switching mechanism in ZnO NW arrays, in which the physical mechanism is shown to be influenced by the presence of a base layer in between the NWs and the growth substrate that actively participates during the switching events[1]. Thus, planar devices based on single isolated NWs are more suitable to investigate the switching mechanism in these nanostructures. Concerning single ZnO NW devices, resistive switching was observed mainly in single isolated NWs contacted by Ti or Ti/Au electrodes[7–10]. In these cases, as revealed by TEM measurements by Chiang et al.[10], a TiO_2 interfacial layer is formed at the Ti/ZnO interface. This layer, that arise from the higher affinity of Ti compared to Zn with O (enthalpies for TiO_2 formation is -944 kJ/mol while for ZnO is -350 kJ/mol)[10], can have a strong influence on the physical mechanism of switching that can be thus localized only at the ZnO/metal interface and not along the NW. Indeed, the switching mechanism can be dominated by the oxygen ion migration at the ZnO-TiO_2 interface, as observed in ZnO/TiO_2 multi-segmented NWs by Huang et al.[15]. A different mechanism of switching in single ZnO NWs was reported in few works by considering electrochemical active electrodes such as Cu[11,12] or Ag[13,14]. In this case, the switching mechanism was explained in terms of metal ion migration along the NW. However, ZnO NW-based devices contacted with a Cu electrode suffer from poor reliability in terms of endurance (only 7 sweep

cycles were reported in a Pd-Au/ZnO NW/Cu device) while very high SET and RESET voltages (> 20 V) were observed in symmetrically contacted NWs[13,14]. All these observations show the importance of the choice of an appropriate device design for the realization of NW-based memristors with controlled switching mechanism and suitable for real applications. In our works, we realized memristive devices by using Ag and Pt asymmetric electrodes. The choice of Pt as counter electrode is crucial since on one hand side it guarantees the absence of an interface layer between the metal/ZnO junction due to the negligible tendency of Pt to form oxides and on the other hand it allows the formation of a Shottky barrier with ZnO that limits the current flowing into the NW when high electric fields are applied. In addition, the high energy required for Pt atoms extraction from the metallic contact makes the electromigration of Pt adatoms on the surface practically not possible.[16] Thus, in our case the switching mechanism can be solely attributed to the redox reactions and movement of Ag ions/atoms from the Ag electrochemically active electrode towards the Pt inert counter electrode.

Supplementary Note 2:

Note that the quantized change of the NW conductivity observed in Fig. 6c can be observed only by reducing the voltage amplitude during the programming pulse. Low voltage pulses result in a slowdown of the conductive path formation kinetic, thus making possible to temporally resolve multiple quantum conductance steps that happen at the same time when faster dynamics are driven by higher voltage pulses.[17,18] This explains why pulses with low amplitude (3V, Fig. 6c) resulted in quantized conductance steps while high voltage pulses (> 5V, Fig. 4a,b) resulted in an abrupt and immediate increase of conductance.

Supplementary References

1. Milano, G. *et al.* Tuning ZnO Nanowire Dissolution by Electron Beam Modification of Surface Wetting Properties. *J. Phys. Chem. C* **122,** 8011–8021 (2018).
2. Cuscó, R. *et al.* Temperature dependence of Raman scattering in ZnO. *Phys. Rev. B* **75,** 165202 (2007).
3. Zhang, R., Yin, P.-G., Wang, N. & Guo, L. Photoluminescence and Raman scattering of ZnO nanorods. *Solid State Sci.* **11,** 865–869 (2009).
4. Milano, G. *et al.* Unravelling Resistive Switching Mechanism in ZnO NW Arrays: The Role of the Polycrystalline Base Layer. *J. Phys. Chem. C* **122,** 866–874 (2018).
5. Chang, W. Y., Lin, C. A., He, J. H. & Wu, T. B. Resistive switching behaviors of ZnO nanorod layers. *Appl. Phys. Lett.* **96,** 242109 (2010).
6. Porro, S. *et al.* Multiple resistive switching in core–shell ZnO nanowires exhibiting tunable surface states. *J. Mater. Chem. C* **5,** 10517–10523 (2017).
7. Zhang, R. *et al.* Enhanced non-volatile resistive switching in suspended single-crystalline ZnO nanowire with controllable multiple states. *Nanotechnology* **27,** 315203 (2016).
8. Lai, Y., Xin, P., Cheng, S., Yu, J. & Zheng, Q. Plasma enhanced multistate storage capability of single ZnO nanowire based memory. *Appl. Phys. Lett.* **106,** 031603 (2015).
9. Lai, Y. *et al.* Resistive Switching of Plasma–Treated Zinc Oxide Nanowires for Resistive Random Access Memory. *Nanomaterials* **6,** 16 (2016).
10. Yen-De Chiang *et al.* Single-ZnO-Nanowire Memory. *IEEE Trans. Electron Devices* **58,** 1735–1740 (2011).
11. Yang, Y. *et al.* Nonvolatile resistive switching in single crystalline ZnO nanowires.

Nanoscale **3,** 1917 (2011).

12. Raffone, F., Risplendi, F. & Cicero, G. A New Theoretical Insight Into ZnO NWs Memristive Behavior. *Nano Lett.* **16,** 2543–2547 (2016).
13. Qi, J. *et al.* Current self-complianced and self-rectifying resistive switching in Ag-electroded single Na-doped ZnO nanowires. *Nanoscale* **5,** 2651 (2013).
14. Wang, B. *et al.* Resistive switching in Ga- and Sb-doped ZnO single nanowire devices. *J. Mater. Chem. C* **3,** 11881–11885 (2015).
15. Huang, C.-W., Chen, J.-Y., Chiu, C.-H. & Wu, W.-W. Revealing Controllable Nanowire Transformation through Cationic Exchange for RRAM Application. *Nano Lett.* **14,** 2759–2763 (2014).
16. Raffone, F. & Cicero, G. Does platinum play a role in the resistance switching of ZnO nanowire-based devices? *Solid State Ionics* **299,** 93–95 (2017).
17. van den Hurk, J., Linn, E., Zhang, H., Waser, R. & Valov, I. Volatile resistance states in electrochemical metallization cells enabling non-destructive readout of complementary resistive switches. *Nanotechnology* **25,** 425202 (2014).
18. Menzel, S., Tappertzhofen, S., Waser, R. & Valov, I. Switching kinetics of electrochemical metallization memory cells. *Phys. Chem. Chem. Phys.* **15,** 6945 (2013).
