# Supplementary material for: Self-limited single nanowire systems combining all-in-one memristive and neuromorphic functionalities
Source: Nat Commun. 2018 Dec 4;9:5151. doi: 10.1038/s41467-018-07330-7 (PMC6279771; doi:10.1038/s41467-018-07330-7)
Supplement: Supplementary file 2 — Peer Review File [file 41467_2018_7330_MOESM2_ESM.pdf]

Editorial Note: This manuscript has been previously reviewed at another journal that is not operating a transparent peer review scheme. This document only contains reviewer comments and rebuttal letters for versions considered at Nature Communications.

REVIEWERS' COMMENTS:

Reviewer #3 (Remarks to the Author):

I have gone through the revised manuscript and response letter and concluded that the authors have addressed my previous questions. Given that a model system as the one demonstrated in the manuscript is very useful for clarifying a number of fundamental issues in memristive systems, I support the publication of this manuscript in Nature Communications.

We thank again the reviewers for reading and commenting on our manuscript.

Reviewer #3 (Remarks to the Author):

I have gone through the revised manuscript and response letter and concluded that the authors have addressed my previous questions. Given that a model system as the one demonstrated in the manuscript is very useful for clarifying a number of fundamental issues in memristive systems, I support the publication of this manuscript in Nature Communications.

R1: We thank the reviewer for the positive recommendation.
